# Reconsidering the role of orthographic redundancy in visual word recognition

**DOI:** 10.3389/fpsyg.2015.00645

**Published:** 2015-05-18

**Authors:** Fabienne Chetail

**Affiliations:** Laboratoire Cognition Langage et Développement, Center for Research in Cognition and Neurosciences, Université Libre de Bruxelles, BruxellesBelgium

**Keywords:** orthographic redundancy, orthographic regularities, bigram frequency, visual word recognition, reading, statistical learning

## Abstract

Humans are known to continuously extract regularities from the flow of stimulation. This occurs in many facets of behavior, including reading. In spite of the ubiquitous evidence that readers become sensitive to orthographic regularities after very little exposure to print, the role of orthographic regularities receives at best a peripheral status in current theories of orthographic processing. In the present article, after the presentation of previous evidence on orthographic redundancy, the hypothesis that orthographic regularities may play a prominent role in word perception is developed.

Humans are known to continuously extract regularities from the flow of stimulation, helping them to perceive the structure of the world, and thus to decrease uncertainty, to repeat successful strategies, and to reduce the information load (Gibson, 1971). How individuals capture regularities is an issue that has led to extensive work, especially in the fields of implicit learning and language acquisition, and despite a substantial number of studies, the way regularities are encoded (extraction of abstract rules or statistical computations) and the function of consciousness in the learning process are still open and hotly debated questions in these fields (see Perruchet and Pacton, 2006).

The continuous extraction of regularities from the flowing array of stimulation occurs in many facets of behavior, including reading. Imagine that you have to choose the most wordlike letter string between *innaro* and *ihharo*, which one would you prefer? And between *kkoxir* and *koxxir*? Pacton et al. (2001) showed that 6-year-old children already manifest a preference for the items you may have also selected. We prefer *innaro* to *ihharo* because *h* is never doubled (at least in languages such as French or English) and *koxxir* to *kkoxir* because, although neither *k* and *x* can be doubled, we know that doubled consonants never occur at word onset. Such facts about letter co-occurrences, or *orthographic regularities*, are not taught but they are part of our implicit language knowledge and they influence the way we perceive written stimuli. Surprisingly, however, despite the general agreement that exposure to print leads to capture orthographic regularities (e.g., Singer, 1980; Cassar and Treiman, 1997; Pacton et al., 2001; Samara and Caravolas, 2014), very little is known about the precise influence of orthographic regularities on word recognition and how it impacts letter string processing. Here, after defining what orthographic regularities are, I present a concise review of the findings. Based on the evidence, the hypothesis that orthographic regularities play a prominent role in word processing is developed.

## What Orthographic Regularities are and are Not

Orthographic regularities refer to facts about the distribution of single letters or letter sequences in print, without direct reference to higher-order levels such as phonological or morphological units (e.g., Henderson and Chard, 1980; Massaro et al., 1981; Seidenberg, 1987). Most of the time, the regularity of appearance of letter clusters is estimated by their absolute or relative frequency of occurrence in written texts. In English for example, the letters *S* and *A* co-occur more frequently in words than the letters *J* and *A*, the letter *R* is more often doubled than the letter *D*, the trigram *CHA* is more frequent at the beginning of words than *PSA*, and the letter *T* is never followed by the letter *X*. In the latter case, illegal letter clusters (e.g., TX) are considered as extreme cases of low frequency *n*-grams (null frequency) and are usually compared with legal *n*-grams. Orthographic redundancy is the general term used to refer to all these slightly different types of orthographic regularities (e.g., Seidenberg, 1987; Andrews, 1992; Conrad et al., 2009). Note, however, that other similar terms have been used to refer to orthographic regularities, such as statistical redundancy (e.g., Massaro et al., 1981), graphotactic or orthotactic regularity (e.g., Pacton et al., 2005), orthographic typicality (e.g., Vinckier et al., 2007), and sequential or spatial frequency (e.g., McClelland and Johnston, 1977). The terms used are sometimes directly constrained by the need to specify which type of regularities is examined (e.g., bigram frequency, for cluster of two letters; trigram frequency, for cluster of three letters) or how they are computed (e.g., positional redundancy, i.e., frequencies computed according to cluster position in words).

It is worth mentioning that measures of orthographic redundancy has been mostly based on the frequency of letter co-occurrences rather than on letter transitional probabilities. In principle, pure letter transitional regularities can be examined in visual word recognition since, for example, although the bigrams GO and HO occur as frequently at the beginning of English words, the probability that the letter G is followed by the letter O is roughly three times higher than the probability that H is followed by O (computations based on the Celex database; Baayen et al., 1993). The early studies on orthographic redundancy manipulated transitional probabilities (usually without strict control of letter co-occurrence frequencies, e.g., Smith, 1969; Rumelhart and Siple, 1974; Butler et al., 1984) but nowadays, the examination of this kind of regularities seems to be restricted to the spoken modality (e.g., Saffran et al., 1996), likely due to the directional nature of the signal, which is absent in written word perception.

Importantly, despite their surface similarity, the concept of ‘orthographic regularities’ is different from the concept of ‘ortho-phonological regularity’ (sometimes shortened in ‘orthogaphic regularity,’ e.g., Mason, 1978a; Seidenberg, 1987). Ortho-phonological regularity refers to the degree of consistency in print-to-sound mapping, mostly examined in terms of variations in the frequency of correspondence between graphemes and phonemes (e.g., Jared et al., 1990; Stone et al., 1997; Hino and Lupker, 2000). For example, in English the grapheme GG almost systematically maps onto the phoneme /g/ (*p* = 0.971), IE frequently maps onto /i/ (*p* = 0.492), and OO rarely maps onto /o/ (*p* = 0.029; Berndt et al., 1987). However, the fact that the grapheme IE more frequently maps onto the phoneme /i/ than onto /ai/ is independent from the frequency of co-occurrence of the letters I and E *per se*. Moreover, the fact that a letter cluster corresponds to a grapheme does not guarantee that it is more recurrent than other non-graphemic letter clusters (e.g., ST is more frequent than SH). The same happens with ortho-morphological mapping. Knowing that TRI frequently maps onto a morpheme referring to threeness (e.g., *triangle*, *triceps* vs. *trial*) provides no information on its frequency compared to the non-morphemic cluster PRI. This kind of independence between orthographic redundancy and ortho-phonological/morphological regularity explains why the phonological and morphological structure of words is not taken into account when defining orthographic regularities. It does not mean that a frequent letter cluster never corresponds to a grapheme or a morpheme, but this is simply not a sine qua non-condition to define orthographic regularities. Importantly, however, given that the natural function of writing is to code speech (and therefore the meaning it conveys), orthographic regularities and ortho-phonological/morphological regularity necessarily overlap to some extent. Thus, if a phoneme (or a morpheme) is frequent in speech and if it is preferentially coded by a given orthographic cluster, then the frequency of this cluster –derived from visual inputs–, is necessarily high, mirroring phonological or morphological regularities present in the lexicon.

## Effects of Orthographic Redundancy: A Review

Following the seminal work of Cattell (1886) on the chronometry of word reading, a line of research tried to precisely identify the factors facilitating the visual perception of words. In these early studies, letter strings were tachistoscopically presented during a brief amount of time and participants had to correctly identify letter strings or to freely report one or several of their letters. Using this method, Miller et al. (1954) were the first to show an influence of orthographic redundancy. When long non-words were presented, the number of letters correctly reported increased when the letter sequences of the non-words more and more approximated letter sequences of English words (e.g., non-words like *vernalist* vs. *ozhgpmtj*). This study paved the way to many other experiments (see **Table 1** for a summary).

**Table 1:** Summary of the main behavioral studies on orthographic redundancy with a direct manipulation of *n*-gram frequency and/or letter transitional probability (ordered by task).

Study	Task	Items	Variable	Direction of the effect
Biederman (1966, Experiment 1)	Letter/word report	Words (same as Owsowitz, 1963)	Bigram frequency (entire string)	Facilitative (LF words)
Biederman (1966, Experiment 2)	Letter/word report	LF words	Bigram frequency (entire string)	Facilitative
Broadbent and Gregory (1968)	Letter/word report	HF and LF words	Bigram frequency (one in the string)	Detrimental (LF words)
Butler et al. (1984, Experiment 1)	Letter/word report	Non-words	Letter transitional probability	Facilitative (stronger effect for high than low-vocabulary readers)
Colegate and Eriksen (1972)	Letter/word report	Trigrams	Letter transitional probability (entire string)	Facilitative
Johnston (1978)	Letter/word report	Words	Summed bigram frequency	No effect (correlations)
Manelis (1974)	Letter/word report	Words, non-words	Summed bigram and trigram frequencies (entire string)	No effect (correlations)
McClelland and Johnston (1977)	Letter/word report	Words, non-words	Mean bigram frequency (entire string)	No effect (but effect of single letter frequency in a *post hoc* analysis)
Miller et al. (1954)	Letter/word report	Non-words	Letter transitional probability	Facilitative
Owsowitz (1963)	Letter/word report	Words	Bigram frequency (entire string)	Detrimental
Rumelhart and Siple (1974)	Letter/word report	Trigrams	Letter transitional probability (entire string)	Facilitative
Scheerer-Neumann (1981)	Letter/word report	Non-words	Letter transitional probability	Facilitative (stronger for good than poor readers)
Smith (1969)	Letter/word report	Words, non-words	Letter transitional probability (entire string)	Facilitative
Spoehr and Smith (1975)	Letter/word report	Words, non-words	Spelling pattern	Facilitative
Baron (1975, Experiment 4)	Letter detection	Words, non-words	Letter sequence legality	Facilitative
Ktori and Pitchford (2009)	Letter detection	Non-words	Single letter frequency	No effects (dyslexic readers)
Mason (1978b, Experiment 2)	Letter detection	Artificial words	Single letter frequency	Facilitative (two extreme conditions)
Massaro et al. (1979)	Letter detection	Non-words	Summed single letter frequency (entire string)	Facilitative
Massaro et al. (1981)	Letter detection	Non-words	Summed bigram frequency (entire string)	Facilitative
Pitchford et al. (2008)	Letter detection	Non-words	Single letter frequency	Facilitative (first and last position within items)
Pitchford et al. (2009)	Letter detection	Non-words	Single letter frequency	Facilitative for the first position (English, Greek), facilitative for the last position in English and detrimental in Greek
Chambers and Forster (1975)	Same-different (simultaneous)	Non-words	Letter sequence legality	Facilitative (positive responses), no effect (negative responses)
Baron (1975, Experiment 2)	Same-different (simultaneous)	Words, non-words	Letter sequence legality	Facilitative
Andrews (1992, Experiment 3)	Lexical decision	HF and LF words, non-words	Summed bigram frequency (entire string)	No effect
Burani and Cafiero (1991, Experiment 2)	Lexical decision	LF words	Initial-consonantal bigram frequency	No effect
Chetail et al. (2014a)	Lexical decision	Words	Mean bigram frequency (entire string)	Detrimental (regressions)
Conrad et al. (2009, Experiment 3)	Lexical decision	Words	First bigram frequency	Facilitative
Gernsbacher (1984, Experiment 3)	Lexical decision	High and low familiar words, non-words	Mean bigram frequency (entire string)	No effects for words, detrimental for non-words
Gernsbacher (1984, Experiment 4)	Lexical decision	High and low familiar words, non-words	Mean bigram frequency (entire string)	No effect
Henderson and Chard (1980)	Lexical decision	Non-words	Letter positional frequency	Detrimental
Keuleers et al. (2012)	Lexical decision	Words	Bigram frequency (summed)	No effect
Rice and Robinson (1975)	Lexical decision	HF and LF words, non-words	Bigram frequency	Detrimental (LF words)
Westbury and Buchanan (2002)	Lexical decision	HF and LF Words, non-words	Frequency of the least frequent bigram	Detrimental (HF words)
Hand et al. (2012)	Sentence reading	Words	First trigram frequency	Detrimental
Lima and Inhoff (1985)	Sentence reading	Words	First trigram frequency	Facilitative
Andrews (1992, Experiment 4)	Naming	HF and LF words	Summed bigram frequency	Facilitative (LF words)
Andrews (1992, Experiment 5)	Naming	HF and LF words	Summed bigram frequency	No effect
Chetail et al. (2014a)	Naming	Words	Mean bigram frequency (entire string)	Detrimental (regressions)
Mason (1978a)	Naming	HF and LF words, non-words	Single letter frequency	Facilitative in non-words
New and Grainger (2011)	Alphabetic decision task	Letters	Single letter frequency	Facilitative

*HF, high frequency; LF, low frequency. The direction of the effects is given when *n*-gram frequency or letter transitional probability increased. The terms ‘letter report’ are used when participants were asked to report letter identity, but the method could vary according to the study. The term ‘non-word’ is used to refer to stimuli that did not correspond to words, independently of their similarity with words (e.g., pronounceable strings, consonantal strings).*

Still with tachistoscopic presentation, Owsowitz (1963) found that the threshold of word identification was lower for both high- and low-frequency words when they entailed bigrams of low rather than high frequency (e.g., *elect* and *beach*, respectively). This effect was opposite to what was expected based on Miller et al. (1954) study, and it has been referred to as the ‘paradoxical bigram effect.’ Broadbent and Gregory (1968) replicated the paradoxical effect, but only for low-frequency words. On the contrary, Biederman (1966) found a facilitative effect of bigram frequency for low-frequency words (and no effect for high-frequency words). This facilitative effect was then replicated by Smith (1969) and by Colegate and Eriksen (1972) who showed a higher proportion of correctly reported letters in stimuli with redundant letter clusters (see also Rumelhart and Siple, 1974; Baron, 1975; Spoehr and Smith, 1975; Scheerer-Neumann, 1981). In the same line, Massaro et al. (1979, 1981) showed that it was easier to detect an individual letter in a word with high letter-redundancy than low letter-redundancy (see also Mason, 1978a). However, other studies failed to observe effects of bigram or trigram frequency in letter or word report (e.g., Manelis, 1974; McClelland and Johnston, 1977; Johnston, 1978).

To bypass the memory task requirements present in free report (Adams, 1981), other tasks have been used to examine the impact of orthographic regularities on word processing. In the same-different task with simultaneous presentation, Chambers and Forster (1975) found that readers were faster to respond ‘same’ for non-words with legal bigrams (e.g., *FOON–FOON*) than with illegal bigrams (e.g., *FT* in *FTRE–FTRE*), which was replicated by Baron (1975).

In the lexical decision task, results consistently showed that non-words with orthographic regularities close to those of real words are harder to reject (e.g., Henderson and Chard, 1980; Gernsbacher, 1984). Regarding words, Rice and Robinson (1975) initially replicated the paradoxical bigram effect for low-frequency words, words with high orthographic redundancy being recognized more slowly than words with low orthographic redundancy. This finding was confirmed more recently by Westbury and Buchanan (2002) for high-frequency words. Conrad et al. (2009), however, found the opposite effect, since words beginning with a frequent bigram were processed more rapidly than words with low-frequency bigrams. Additionally, some studies found no significant effect of bigram frequency in high- or low-frequency words in the lexical decision task (Burani and Cafiero, 1991; Andrews, 1992; Keuleers et al., 2012). However, when the effect was examined through regression analyses based on megastudies (inclusion of more than 5,000 items and control of many variables), a highly reliable inhibitory effect of bigram frequency was found (Chetail et al., 2014a).

In the naming task, Mason (1978a) reported that non-words with high single letter frequency were named more rapidly than those with low letter frequency, but no effect was found in words. On the contrary, Andrews (1992) initially showed that words with bigrams of high frequency were named more quickly than those with bigrams of low-frequency, but this was finally explained in terms of confound with the first phoneme identity. However, regression analyses based on megastudies yielded an inhibitory effect of bigram frequency, even after the effect of first phoneme was controlled (Chetail et al., 2014a).

Finally, in the sentence reading task combined with eye movement recording, Lima and Inhoff (1985) initially reported a facilitative effect of trigram frequency, although only on first fixation durations. A follow-up study conducted by Hand et al. (2012) reported, however, a detrimental effect of trigram frequency. Because the effect was consistently found on a large range of eye movement measures (including first and single fixation durations, gaze duration, and total fixation time), the authors considered their results more reliable than those of Lima and Inhoff (1985), and they explained the discrepancy in terms of materials and method differences (e.g., number of word neighbors; number of data points; length of items preceding target words).

## Understanding the Effects: Different Roles of Orthographic Redundancy

This review of empirical findings shows a mixed picture of orthographic redundancy effects, with some studies reporting facilitative effects (e.g., Biederman, 1966; Massaro et al., 1981; Pitchford et al., 2008; Conrad et al., 2009), others showing detrimental effects (e.g., Broadbent and Gregory, 1968; Rice and Robinson, 1975; Westbury and Buchanan, 2002; Chetail et al., 2014a), and still others leading to null effects (e.g., Johnston, 1978; Burani and Cafiero, 1991; Andrews, 1992). Moreover, orthographic redundancy was considered at different grain sizes (single letters, bigrams, trigrams), leading sometimes to inconsistent results (e.g., McClelland and Johnston, 1977). The proposal developed here is that such inconsistency is due to the fact that orthographic regularities play different roles during written word processing, from the analysis of stimulus input to word meaning access. The idea that sensitivity to orthographic redundancy has a *utility* for written word processing and that readers employ their tacit knowledge of orthographic regularities to process letters strings emerged decades ago (Miller et al., 1954; Estes, 1975; Henderson and Chard, 1980; Adams, 1981), but different hypotheses can be put forward to explain the precise role of orthographic regularities. Some of these hypotheses were considered in the past, from time to time, separately, but usually without taking into account the whole evidence available (or lack of evidence) and without being discussed together. This is the aim of the present section.

*A first hypothesis* is that orthographic redundancy facilitates the identification of letters in the very first steps of written word perception. Support for this assumption comes from the evidence that efficiency of letter perception increases with single letter frequency (e.g., Mason, 1978b; Massaro et al., 1979; New and Grainger, 2011). For example, New and Grainger (2011) reported that the time required to decide whether a symbol is a letter or not decreases with single letter frequency. This facilitation is assumed to stem from the activation of letter features, with an increase of the firing strength of neurons coding for features present in frequent letters or from an increase of synchronization in the firing within a particular ensemble of neurons (see Gilbert et al., 2001). Consistently, recent modeling with an interactive activation (IA) model with localist representations showed that the best account for EEG output associated to letter perception was found when feature-to-letter excitatory connections, lateral letter inhibition, and letter-to-feature feedback were implemented (Rey et al., 2009).

*A second function* of orthographic regularities would be to help readers to encode the order of letters in strings. The idea that the perception of letter position can be noisy during written word processing was discussed as soon as the 1970s (e.g., Estes, 1975; Estes et al., 1976) and came from the observation that pseudowords built from words by a transposition of two adjacent letters (e.g., *gadren* from *garden*) were frequently misperceived as their corresponding base words (e.g., Bruner and O’Dowd, 1958; see also Chambers, 1979). This has gradually led to the development of models of orthographic processing that implemented a flexible encoding of letter position (e.g., Grainger and Van Heuven, 2003; Davis and Bowers, 2006; Gomez et al., 2008; Davis, 2010). Letter position coding in these models fully rely on the *identity* of letters, so they predict identical outputs (be it measured in number of open bigrams, Grainger and Van Heuven, 2003; spatial coding scheme; Davis, 2010; or degree of perceptual overlap, Gomez et al., 2008) when the characteristics of transposed letters (letter frequency, consonant-vowel status) are different and influence adjacent letters (leading to illegal bigrams, Perea and Carreiras, 2008; or to changes in the CV pattern of words, Chetail et al., 2014b).

However, several authors in the 1970s proposed that transposed letter effects and orthographic redundancy are tightly linked: One fundamental function of orthographic regularities would be to counteract weak information of letter position (Estes et al., 1976; Katz, 1977; Adams, 1979). First supporting evidence for this hypothesis came from the fact that the percentage of transposed letters was higher for bigrams with the lowest frequency in the letter report task (Estes et al., 1976). More recently, Perea and Carreiras (2008) examined the effect of transposed bigram legality in the primed lexical decision task (SOA = 50 ms). They found that primes such as *comsos* (illegal transposed bigram *MS*, in Spanish) facilitated the processing of *COSMOS* as well as an identity prime, contrary to primes such as *vebral* for *VERBAL* that include a legal transposed bigram (BR). In other words, priming a bigram with an illegal transposed letter bigram (e.g., *ms – SM*) yielded a stronger effect than priming a bigram with a legal transposed bigram (e.g., *br – RB*; see also Frankish and Barnes, 2008). Perea and Carreiras (2008) proposed that during lexical access, illegal bigrams would be normalized into legal bigrams (e.g., *ms* →*sm* priming *SM*), increasing the priming effect compared to legal bigrams which are not normalized (e.g., *br* priming *RB*). Illegal bigrams being extreme cases of low bigram frequency, this account is compatible with Estes et al. (1976)’s finding that participants are more prone to normalize (i.e., transpose) low-frequency bigrams than high-frequency bigrams in free letter report.

Since the analysis of the letter strings initially leads to the activation of letter feature and letter representations (see Grainger et al., 2008), facilitation in the very first steps of visual word recognition (letter identification and letter position encoding) could be driven by frequencies of *single letters* rather than by local *n*-grams. Consistently, a facilitative effect of orthographic redundancy was systematically reported when single letter frequency was manipulated in tasks focusing on letter processing (see **Table 1**). Moreover, despite a null effect of bigram frequency, McClelland and Johnston (1977) found that the letters with the highest frequencies at a given position led to a higher accuracy of letter report.

Regarding letter position coding, the shape of the entire distribution of positional single letter frequencies would even be critical (Katz, 1977). The positional distribution of letters refers to the fact that a letter can either occur roughly as frequently at all or almost all positions within words (e.g., the letter R in English appears quite uniformly at the five positions of five-letter words) or it can occur more predominantly at one or two positions only (e.g., the letter H appears the most frequently at the second position and the least frequently at the third one). The second type of distribution (peaked distribution) may be the most informative to reduce letter position uncertainty because it specifies at which position a letter is likely (or unlikely) to occur. Katz (1977) actually showed that adults –as well as good readers in grade 5– had knowledge of the distributions of letter positions and used them to perform a forced-choice task during which they had to choose the most frequent position at which a given letter occurs. Consistently, Pitchford et al. (2008) and Ktori and Pitchford (2009) showed that readers were more rapid to detect letters at their most frequent position, especially in the first and last positions.

*A third hypothetical role* of orthographic redundancy is related to the definition of word structure. The starting point of this hypothesis comes from the observation that bigram frequency is generally higher within graphosyllables (ie., letter clusters mapping onto syllables) than between them. This particular pattern is referred to as a bigram trough (Seidenberg, 1987). Orthographic regularities would therefore highlight letter groups that frequently co-occur and would create perceptual boundaries within letter strings (Adams, 1981). For example, the fact that the bigrams *AN* and *VI* are more frequent than *NV* would define a two-parts structure in *ANVIL* with a boundary falling between the letters *N* and *V*. The same logic applied in morphologically complex words like *REUNION*, since bigram frequencies also tend to be higher within morphemes than between them, leading to an orthographic boundary between the letters E and U (Rastle et al., 2004). Hence, according to some authors, orthographic redundancy would be determinant in visual word perception because it makes the structure of words emerge, reflecting regularities that are encoded in the lexicon rather than the activation of sublexical representations such as graphosyllables or morphemes (e.g., Seidenberg, 1987; Seidenberg and McClelland, 1989). For other ones, orthographic redundancy simply facilitates the contact between letter clusters and existing representations of phonological or morphemic units (e.g., Mathey et al., 2006; Grainger and Ziegler, 2011). Despite different frameworks, the point of these two proposals is that, at a certain level of processing, orthographic redundancy facilitates access to the phonological and morphemic structure of words.

Critically, orthographic regularities capture not only the mere letter occurrences or co-occurrences within a lexicon, but also the linguistic structure of words. The nature and grain size of regularities that facilitate the perception of the internal structure of words should therefore be directly determined by the characteristics of mapping between orthography and phonology/morphology in a given language. In French or English for example, the frequency of bigram and trigram clusters may be particularly critical (i.e., more than single letter frequency), because linguistic units most frequently map onto clusters of two or three letters (e.g., affixes usually correspond to bigrams or trigrams). Furthermore, the fact that there are more grapheme-to-phoneme correspondences at the level of bigrams than trigrams such as AE→/i/ vs. EOU →/ә/ in English (e.g., 98 vs. 13 based on Berndt et al., 1987; 103 vs. 85 in French based on Peereman and Content, 1999) and that bigrams most frequently correspond to syllables than trigrams (e.g., 336,303 vs. 244,287 occurrences per million in French, based on Chetail and Mathey, 2010) may support the idea that the bigram grain-size is more critical than the trigram grain-size.

Support for orthographic redundancy as determining word structure comes from the fact that syllable-like effects were found when the boundary between graphosyllables is marked by a bigram trough pattern in letter color detection tasks (e.g., Seidenberg, 1987) and decreased or vanished in the absence of bigram trough (e.g., Doignon and Zagar, 2005, 2006). However, follow-up studies with the lexical decision task reported syllabic or morphemic effects even when there was no bigram trough at the critical boundaries or when this factor was controlled across conditions (e.g., Duñabeitia et al., 2007; Conrad et al., 2009; Muncer and Knight, 2012; Muncer et al., 2014). First, this suggests that orthographic redundancy is not the only clue that influences the perception of word structure. Actually, Chetail and Content recently showed that the way consonant and vowel letters are organized is another clue that strongly determines word perceptual structure (e.g., Chetail and Content, 2012; Chetail et al., 2014b). Second, the absence of bigram trough effects was reported with the lexical decision task, that is, with a task supposed to engage more processes of lexical selection than a letter detection task. Null effects could therefore be explained by the fact that orthographic redundancy does not play a facilitative role during lexical access.

In models based on the IA hypothesis (McClelland and Rumelhart, 1981), recognizing a word implies to activate a lexical representation in the mental lexicon (lexical access), leading to a competition between orthographic representations of similar words (i.e., orthographic neighbors, see Andrews, 1997; Davis et al., 2009, for reviews). *A fourth hypothesis* regarding the role of orthographic redundancy is that it helps to reduce the set of lexical competitors during lexical access. This proposal is supported by the paradoxical bigram effect, mostly reported in tasks requiring lexical access or word report: Words with low-frequency bigrams are processed more efficiently than those with high-frequency bigrams (e.g., Owsowitz, 1963; Broadbent and Gregory, 1968; Rice and Robinson, 1975; Westbury and Buchanan, 2002; Hand et al., 2012; Chetail et al., 2014a). This effect is assumed to occur because low-frequency bigrams are the most constraining (or diagnostic) to identify a word (e.g., Rice and Robinson, 1975; Grainger and Ziegler, 2011). For example, the presence of the bigram EB at the beginning of a word strongly constrains the set of possible words to identify (only nine English words begin with EB) contrary to the bigram EN (315 words).

This interpretation naturally raises questions about the independency of orthographic neighborhood and orthographic redundancy effects. Actually, although measures of orthographic redundancy tend to be correlated with orthographic neighborhood size, neighborhood density effects were found when bigram frequency was matched (e.g., Andrews, 1992; Peereman and Content, 1995), and conversely, bigram frequency effects were reported while orthographic neighborhood density was controlled (e.g., Westbury and Buchanan, 2002; Chetail et al., 2014a). Effects of orthographic redundancy (as estimated by bigram frequency) in the lexical decision task therefore do not appear to be a mere by-product of orthographic neighborhood density (and vice versa).

*A fifth role* of orthographic redundancy is related to bilingual language detection. As monolinguals, bilinguals seem to be sensitive to orthographic regularities of their languages (e.g., bigram trough situations-like, Lemhöfer et al., 2011; see also Frenck-Mestre, 1993). Contrary to monolinguals, however, bilinguals need to quickly identify to which language the written words they read refer to. Among the different cues available, the legality of bigrams in both orthographies would be a reliable marker. For example, Vaid and Frenck-Mestre (2002) tested French-English bilinguals to examine the processing of marked words (i.e., words containing legal or highly frequent bigrams in only one of the two languages, e.g., *eyes* in English, *oeuf* in French) and unmarked (or neutral) words (i.e., words containing bigrams that are legal or frequent in both languages, e.g., *ounce* in English, *aussi* in French). In a speeded language recognition task (i.e., to decide whether items belong to L1 or L2), marked items were processed faster than neutral items, especially for L2 words (see also van Kesteren et al., 2012). This occurs whatever the proficiency of readers in L2, that is, be it balanced bilinguals, less efficient bilinguals in L2, or monolinguals with no knowledge in L2 (Casaponsa et al., 2014).

However, the facilitation of language-specific orthography in the language recognition task seems to ensue from a language decision advantage rather than from a faster word recognition. Indeed, when the status of the bigrams is no more diagnostic for the task (e.g., to do a L2 lexical decision task; i.e., response ‘yes’ for L2 words, response ‘no’ for L1 words and for non-words devised with marked and unmarked bigrams of L2), the markedness effect for L2 words vanished while the effect is present for L1 words (van Kesteren et al., 2012). In the same line, when the participants performed a progressive demasking task (i.e., to identify letter strings with gradual increase of presentation duration), balanced bilinguals had a similar performance in L1 and L2 words and no markedness effect, whereas the unbalanced bilinguals were slower and less accurate for L2 words than for L1 words. When the main aim of the task is not a language membership assignment, participants therefore seem to little rely on sublexical knowledge in favor of lexical knowledge (Casaponsa et al., 2014).

It is worth mentioning that although this line of research clearly shows that bilinguals are sensitive to the legality of bigrams in their two languages and use this information for bilingual language detection, these studies are not directly comparable with previous experiments in monolinguals (see What Orthographic Regularities are and are not). Indeed, the fact that a bigram is specific to a given language (e.g., *sj* existing in Norwegian but not in English) gives a priori no information on the frequency of this bigram in the target orthography. Hence, these studies do not tell us whether bilinguals are sensitive to orthographic regularities (such as bigram frequency) within each orthography and use them during L1 word recognition on the one hand, and L2 word recognition on the other hand.

To summarize Section “Understanding the Effects: Different Roles of Orthographic Redundancy,” orthographic redundancy (especially at the single letter level) may facilitate letter identification and letter position encoding, explaining the presence of facilitative effects in letter report or detection tasks. Redundancy of larger letter clusters (bigrams, trigrams) may also help the perceptual system to process the internal structure of words, due to the recurrent contact between *n*-grams and linguistic units. At later levels of word processing, orthographic regularities may produce a detrimental effect, low-frequency clusters being more discriminant to access word identity. An analysis of the time course of the effects of orthographic redundancy in event related potentials should therefore show early facilitation of orthographic redundancy followed by detrimental effects. Consistently, Hauk et al. (2006) reported that words with high-frequency bigrams and trigrams elicited smaller amplitudes around 110 ms after word onset (which was interpreted as a facilitative effect of orthographic redundancy) and the effect reversed around 280 ms, which can be interpreted as a diagnosticity effect (i.e., words with frequent letter clusters are processed less efficiently).

The fact that orthographic redundancy leads to facilitative and detrimental effects at different levels of processing implies that word characteristics (e.g., large vs. small neighborhood density) and task demands (driven by lexical vs. sublexical processing) should modulate the weight of the sublexical facilitation and the lexical access slowing down due to orthographic regularities. Likely variations of these parameters in previous studies could explain why, for example, the paradoxical bigram effect was not systematically observed (see **Table 1**). More generally, a critical point regarding orthographic redundancy is that it naturally covaries with other factors known to impact visual word recognition, such as pronunceability (Binder et al., 2006), word frequency (Adams, 1981), or orthographic neighborhood (Andrews, 1992). Absence or insufficient matching on these factors can also explain some remaining inconsistencies across studies (e.g., Gernsbacher, 1984; Hand et al., 2012).

## Concluding Remarks and Future Directions

The field of visual word recognition is currently marked by the rapid development of sophisticated models of orthographic processing of words (see Davis and Bowers, 2006; Lerner et al., 2014, for reviews), with minimal consideration for the linguistic structure of lexicons (e.g., Frost, 2012; Taft and Krebs-Lazendic, 2013; Chetail et al., 2014b) and no implementation of orthographic redundancy coding. Some connectionist models actually use algorithms that capture in the end regularities (e.g., Seidenberg and McClelland, 1989; Harm and Seidenberg, 2004), but these models are currently devoted to orthographic mapping onto phonology and semantics and “ignore the development of knowledge concerning the sequential structure of written language, that is, orthographic redundancy” (Harm and Seidenberg, 2004, p. 714). This lack of consideration of orthographic redundancy in models is likely due to the apparent mixed findings reported in the past. The present review demonstrates that results are not as inconsistent as they seem, and that on the contrary, sensitivity to orthographic regularities may influence visual word recognition at all levels of processing.

The sensitivity to orthographic regularities develops very rapidly throughout exposure to print. Both children in late kindergarten exposed to written words of their own language (Cassar and Treiman, 1997; Pacton et al., 2001) and adults exposed few hours to a new script made of artificial symbols representing letters (Singer, 1980) favored letter strings entailing the regularities they learnt. Aside from constraining and modeling the hypotheses on the role of orthographic redundancy in visual word recognition (see Section Summarizing Understanding the Effects: Different Roles of Orthographic Redundancy), future studies will need to link the end state of orthographic redundancy learning to the *development* of the sensitivity to these regularities. This presupposes examining the dynamic of the learning process throughout exposure to print, as a function of the characteristics of the materials to be learnt and of the learners.

Since the characteristics of the printed corpus to which individuals are exposed vary according to the linguistic structure of the language, the nature of orthographic regularities captured by readers is necessarily determined in part by linguistic variables. Indeed, orthographic regularities not only reflect pure statistical redundancy of letter groups, but they also capture the linguistic structure of words, such as their phonological and morphemic form. Thus, in a language with a one-to-one sound-to-print mapping and with many consonant-vowel syllabic structures, the sensitivity to bigram frequencies captures the distribution of letter co-occurrences, the redundancy of consonant-vowel syllables in speech, and the fact that these syllables are consistently coded by specific letter clusters. Hence, not only orthographic redundancy should participate to the activation of word structure, but also linguistic structure of words should fine-tunes and reinforces the types of regularities that are learnt. However, although the structure of the lexicon and the print-to-sound mapping may play a central role in *what is learnt*, the processes underlying the learning process *per se* (*how it is learnt*) are assumed to be similar whatever the language and to be underpinned by a neurobiological network active in the learning of statistical information in different types of stimuli and modalities (see Frost et al., 2015). This leads to consider cross-linguistic differences in orthographic redundancy effects at a general level of description rather than in assuming specific processing according to languages (see Frost, 2012; Lerner et al., 2014 for discussions on a general level of description of cross-linguistic differences).

Regarding the learners’ characteristics, several studies showed that readers vary in their sensitivity to orthographic regularities. Especially, poorer readers tend to exhibit weaker effects of orthographic redundancy than good readers (e.g., Mason, 1975; Adams, 1981; Scheerer-Neumann, 1981; Butler et al., 1984; see also Pitchford et al., 2009), but it is not clear whether this reflects an inadequate knowledge of sequential redundancy from poor readers or their inability to use their knowledge as an additional source of information (Mason, 1975), or even a mere lack of print exposure. Addressing issues on the individual ability to extract and use orthographic regularities will be particularly helpful to understand what differentiates poor and good readers and what underlies reading efficiency. Indeed, as hypothesized by Nation et al. (2007), the level of sensitivity to orthographic regularities may be a strong predictor of the ability to precisely encode orthographic information of new words and is related to a general capacity of reading. This assumption is supported by the finding that the general capacity to extract statistical regularities in sequences of shapes is correlated to the efficiency of learning a new language, measured by naming accuracy (Frost et al., 2013). If one assumes that learning to read in a new language requires individuals to acquire a new lexical system mainly by picking up and assimilating a set of specific statistical regularities (Frost et al., 2013), the degree of sensitivity to statistical properties of printed words should predict reading efficiency.

The view that the characteristics of both learners and materials to be learnt need to be considered to fully account for the role of orthographic redundancy is in line with the more general claim that understanding the source of inter-individual differences is a keystone for understanding the mechanism of statistical properties learning (Frost et al., 2015). Frost et al. (2015) assumed two main sources to the variance across individuals in statistical learning; one related to the efficiency in encoding representations (testable in manipulating encoding parameters, such as stimuli complexity or display duration of stimuli), and one related to the efficiency in registering statistical properties (testable in manipulating for example the type of statistical contingencies present in the stimuli). In the frame of orthographic regularities learning, disentangling these two types of variance may be promising to define the parameters that enable the reading system to reach a stable state of knowledge on orthographic redundancy and to use the learning output to process words in familiar and less familiar orthographies.

## Conflict of Interest Statement

The author declares that the research was conducted in the absence of any commercial or financial relationships that could be construed as a potential conflict of interest.

## References

[B1] Adams M. J. (1979). Models of word recognition. *Cogn. Psychol.* 11 133–176 10.1016/0010-0285(79)90008-2

[B2] Adams M. J. (1981). “What good is orthographic redundancy?” in *Perception of Print: Reading Research in Experimental Psychology*, eds Tzeng O. J. L. Singer H. (Hillsdale, MI: Lawrence Erlbaum Associates), 197–221.

[B3] Andrews S. (1992). Frequency and neighborhood effects on lexical access: lexical similarity or orthographic redundancy? *J. Exp. Psychol. Learn. Mem. Cogn.* 18 234–254 10.1037/0278-7393.18.2.234

[B4] Andrews S. (1997). The effect of orthographic similarity on lexical retrieval: resolving neighborhood conflicts. *Psychon. Bull. Rev.* 4 439–461 10.3758/BF03214334

[B5] Baayen R. H. Piepenbrock R. van Rijn H. (1993). *The CELEX Lexical Database* (*CD ROM*). Philadelphia, PA: Linguistic Data Consortium, University of Pennsylvania.

[B6] Baron J. (1975). “Successive stages in word recognition,” in *Attention and Performance V*, eds Domic S. Rabbitt P. M. A. (New York, NY: Academic Press), 563–574.

[B7] Berndt R. S. Reggia J. A. Mitchum C. C. (1987). Empirically derived probabilities for grapheme-to-phoneme correspondences in english. *Behav. Res. Methods Instrum. Comput.* 19 1–9 10.3758/BF03207663

[B8] Biederman G. B. (1966). Supplementary report: the recognition of tachistoscopically presented five-letter words as a function of digram frequency. *J. Verbal Learn. Verbal Behav.* 5 208–209 10.1016/S0022-5371(66)80020-8

[B9] Binder J. R. Medler D. A. Westbury C. F. Liebenthal E. Buchanan L. (2006). Tuning of the human left fusiform gyrus to sublexical orthographic structure. *Neuroimage* 33 739–748 10.1016/j.neuroimage.2006.06.053 16956773 PMC1634933

[B10] Broadbent D. E. Gregory M. (1968). Visual perception of words differing in letter digram frequency. *J. Verbal Learn. Verbal Behav.* 7 569–571 10.1016/S0022-5371(68)80052-0

[B11] Bruner J. S. O’Dowd D. (1958). A note on the informativeness of parts of words. *Lang. Speech* 1 98–101 10.1177/002383095800100203

[B12] Burani C. Cafiero R. (1991). The role of subsyllabic structure in lexical access to printed words. *Psychol. Res.* 53 42–52 10.1007/BF00867331

[B13] Butler B. E. Jared D. Hains S. (1984). Reading skill and the use of orthographic knowledge by mature readers. *Psychol. Res.* 46 337–353 10.1007/BF00309068

[B14] Casaponsa A. Carreiras M. Duñabeitia J. A. (2014). Discriminating languages in bilingual contexts: the impact of orthographic markedness. *Front. Psychol. Sci.* 5:424 10.3389/fpsyg.2014.00424 PMC4026679 24860536

[B15] Cassar M. Treiman R. (1997). The beginnings of orthographic knowledge: children’s knowledge of double letters in words. *J. Educ. Psychol.* 89 631–644 10.1037/0022-0663.89.4.631

[B16] Cattell J. M. (1886). The time it takes to see and name objects. *Mind* 11 63–65 10.1093/mind/os-XI.41.63

[B17] Chambers S. M. (1979). Letter and order information in lexical access. *J. Verbal Learn. Verbal Behav.* 18 225–241 10.1016/S0022-5371(79)90136-1

[B18] Chambers S. M. Forster K. I. (1975). Evidence for lexical access in a simultaneous matching task. *Mem. Cogn.* 3 549–559 10.3758/BF03197530 24203880

[B19] Chetail F. Balota D. Treiman R. Content A. (2014a). What can megastudies tell us about the orthographic structure of English words? *Q. J. Exp. Psychol.* 10.1080/17470218.2014.963628 [Epub ahead of print]. 25208136

[B20] Chetail F. Drabs V. Content A. (2014b). The role of consonant/vowel organization in perceptual discrimination. *J. Exp. Psychol. Learn. Mem. Cogn.* 40 938–961 10.1037/a0036166 24749964

[B21] Chetail F. Content A. (2012). The internal structure of chaos: letter category determines visual word perceptual units. *J. Mem. Lang.* 67 371–388 10.1016/j.jml.2012.07.004

[B22] Chetail F. Mathey S. (2010). InfoSyll: a syllabary providing statistical information on phonological and orthographic syllables. *J. Psycholinguist. Res.* 39 485–504 10.1007/s10936-009-9146-y 20037781

[B23] Colegate R. L. Eriksen C. W. (1972). Form of redundancy as a determinant of tachistoscopic word recognition. *Percept. Psychophys.* 12 477–481 10.3758/BF03210939

[B24] Conrad M. Carreiras M. Tamm S. Jacobs A. M. (2009). Syllables and bigrams: orthographic redundancy and syllabic units affect visual word recognition at different processing levels. *J. Exp. Psychol. Hum. Percept. Perform.* 35 461–479 10.1037/a0013480 19331501

[B25] Davis C. J. (2010). The spatial coding model of visual word identification. *Psychol. Rev.* 117 713–758 10.1037/a0019738 20658851

[B26] Davis C. J. Bowers J. S. (2006). Contrasting five different theories of letter position coding: evidence from orthographic similarity effects. *J. Exp. Psychol. Hum. Percept. Perform.* 32 535–557 10.1037/0096-1523.32.3.535 16822123

[B27] Davis C. J. Perea M. Acha J. (2009). Re(de)fining the orthographic neighborhood: the role of addition and deletion neighbors in lexical decision and reading. *J. Exp. Psychol. Hum. Percept. Perform.* 35 1550–1570 10.1037/a0014253 19803656

[B28] Doignon N. Zagar D. (2005). Illusory conjunctions in French: the nature of sublexical units in visual word recognition. *Lang. Cogn. Process.* 20 443–464 10.1080/01690960444000269

[B29] Doignon N. Zagar D. (2006). Les enfants en cours d’apprentissage de la lecture perçoivent-ils la syllabe à l’écrit? *Can. J. Exp. Psychol.* 60 258–274 10.1037/cjep2006024

[B30] Duñabeitia J. A. Perea M. Carreiras M. (2007). Do transposed-letter similarity effects occur at a morpheme level? Evidence for morpho-orthographic decomposition. *Cognition* 105 691–703 10.1016/j.cognition.2006.12.001 17217942

[B31] Estes W. K. (1975). “Memory, perception, and decision in letter identification,” in *Information Processing and Cognition*, ed.Solso R. L. (Hillsdale: Lawrence Erlbaum Associates), 3–30.

[B32] Estes W. K. Allmeyer D. H. Reder S. M. (1976). Serial position functions for letter identification at brief and extended exposure durations. *Percept. Psychophys.* 19 1–15 10.3758/BF03199379

[B33] Frankish C. Barnes L. (2008). Lexical and sublexical processes in the perception of transposed-letter anagrams. *Q. J. Exp. Psychol.* 61 381–391 10.1080/17470210701664880 17935005

[B34] Frenck-Mestre C. (1993). Use of orthographic redundancies and word identification speed in bilinguals. *J. Psycholinguist. Res.* 22 397–409 10.1007/BF01074343

[B35] Frost R. (2012). Towards a universal model of reading. *Behav. Brain Sci.* 35 263–279 10.1017/S0140525X11001841 22929057 PMC3677812

[B36] Frost R. Armstrong B. C. Siegelman N. Christiansen M. H. (2015). Domain generality versus modality specificity: the paradox of statistical learning. *Trends Cogn. Sci.* 19 117–125 10.1016/j.tics.2014.12.010 25631249 PMC4348214

[B37] Frost R. Siegelman N. Narkiss A. Afek L. (2013). What predicts successful literacy acquisition in a second language? *Psychol. Sci.* 24 1243–1252 10.1177/0956797612472207 23698615 PMC3713085

[B38] Gernsbacher M. A. (1984). Resolving 20 years of inconsistent interactions between lexical familiarity and orthography, concreteness, and polysemy. *J. Exp. Psychol. Gen.* 113 256–281 10.1037/h0057955 6242753 PMC4311894

[B39] Gibson E. J. (1971). Perceptual learning and the theory of word perception. *Cogn. Psychol.* 2 351–368 10.1016/0010-0285(71)90020-X

[B40] Gilbert C. D. Sigman M. Crist R. E. (2001). The neural basis of perceptual learning. *Neuron* 31 681–697 10.1016/S0896-6273(01)00424-X 11567610

[B41] Gomez P. Ratcliff R. Perea M. (2008). The overlap model: a model of letter position coding. *Psychol. Rev.* 115 577–600 10.1037/a0012667 18729592 PMC2597794

[B42] Grainger J. Rey A. Dufau S. (2008). Letter perception: from pixels to pandemonium. *Trends Cogn. Sci.* 12 381–387 10.1016/j.tics.2008.06.006 18760658

[B43] Grainger J. Van Heuven W. (2003). “Modeling letter position coding in printed word perception,” in *The Mental Lexicon*, ed.Bonin P. (New York, NY: Nova Science Publishers), 1–24.

[B44] Grainger J. Ziegler J. C. (2011). A dual-route approach to orthographic processing. *Front. Psychol.* 2:54 10.3389/fpsyg.2011.00054 PMC3110785 21716577

[B45] Hand C. J. O’Donnell P. J. Sereno S. C. (2012). Word-initial letters influence fixation durations during fluent reading. *Front. Psychol. Sci.* 3:85 10.3389/fpsyg.2012.00085 PMC3317262 22485100

[B46] Harm M. W. Seidenberg M. S. (2004). Computing the meanings of words in reading: cooperative division of labor between visual and phonological processes. *Psychol. Rev.* 111 662–720 10.1037/0033-295X.111.3.662 15250780

[B47] Hauk O. Patterson K. Woollams A. Watling L. Pulvermüller F. Rogers T. T. (2006). [Q:] When would you prefer a SOSSAGE to a SAUSAGE? [A:] At about 100 msec. ERP correlates of orthographic typicality and lexicality in written word recognition. *J. Cogn. Neurosci.* 18 818–832 10.1162/jocn.2006.18.5.818 16768380

[B48] Henderson L. Chard J. (1980). “The reader implicit knowledge of orthographic structure,” in *Cognitive Processes in Spelling*, ed.Frith U. (New York, NY: Academic Press), 85–116.

[B49] Hino Y. Lupker S. J. (2000). Effects of word frequency and spelling-to-sound regularity in naming with and without preceding lexical decision. *J. Exp. Psychol. Hum. Percept. Perform.* 26 166–183 10.1037/h0080739 10696612

[B50] Jared D. McRae K. Seidenberg M. S. (1990). The basis of consistency effects in word naming. *J. Mem. Lang.* 29 687–715 10.1016/0749-596X(90)90044-Z

[B51] Johnston J. C. (1978). A test of the sophisticated guessing theory of word perception. *Cogn. Psychol.* 10 123–153 10.1016/0010-0285(78)90011-7 668297

[B52] Katz L. (1977). Reading ability and single-letter orthographic redundancy. *J. Educ. Psychol.* 69 653–659 10.1037/0022-0663.69.6.653

[B53] Keuleers E. Lacey P. Rastle K. Brysbaert M. (2012). The British Lexicon Project: lexical decision data for 28730 monosyllabic and disyllabic English words. *Behav. Res. Methods* 44 287–304 10.3758/s13428-011-0118-4 21720920 PMC3278621

[B54] Ktori M. Pitchford N. J. (2009). Development of letter position processing: effects of age and orthographic transparency. *J. Res. Read.* 32 180–198 10.1111/j.1467-9817.2009.01394.x

[B55] Lemhöfer K. Koester D. Schreuder R. (2011). When bicycle pump is harder to read than bicycle bell: effects of parsing cues in first and second language compound reading. *Psychon. Bull. Rev.* 18 364–370 10.3758/s13423-010-0044-y 21327384 PMC3070879

[B56] Lerner I. Armstrong B. C. Frost R. (2014). What can we learn from learning models about sensitivity to letter-order in visual word recognition? *J. Mem. Lang.* 77 40–58 10.1016/j.jml.2014.09.002 25431521 PMC4242428

[B57] Lima S. D. Inhoff A. W. (1985). Lexical access during eye fixations in reading: effects of word-initial letter sequence. *J. Exp. Psychol. Hum. Percept. Perform.* 11 272–285 10.1037/0096-1523.11.3.272 3159838

[B58] Manelis L. (1974). The effect of meaningfulness in tachistoscopic word perception. *Percept. Psychophys.* 16 182–192 10.3758/BF03203272

[B59] Mason M. (1975). Reading ability and letter search time: effects of orthographic structure defined by single-letter positional frequency. *J. Exp. Psychol. Gen.* 104 146–166 10.1037/0096-3445.104.2.146

[B60] Mason M. (1978a). From print to sound in mature readers as a function of reader ability and two forms of orthographic regularity. *Mem. Cogn.* 6 568–581 10.3758/BF03198246 24203391

[B61] Mason M. (1978b). The role of spatial redundancy in grapheme recognition: perception or inference? *J. Exp. Psychol. Hum. Percept. Perform.* 4 662–673 10.1037/0096-1523.4.4.662 722254

[B62] Massaro D. W. Jastrzembski J. E. Lucas P. A. (1981). Frequency, orthographic regularity, and lexical status in letter and word perception. *Psychol. Learn. Motiv.* 15 163–200 10.1016/S0079-7421(08)60175-9

[B63] Massaro D. W. Venezky R. L. Taylor G. A. (1979). Orthographic regularity, positional frequency, and visual processing of letter strings. *J. Exp. Psychol. Gen.* 108 107–124 10.1037/0096-3445.108.1.107 528895

[B64] Mathey S. Zagar D. Doignon N. Seigneuric A. (2006). The nature of the syllabic neighbourhood effect in French. *Acta Psychol.* 123 372–393 10.1016/j.actpsy.2006.02.003 16620742

[B65] McClelland J. L. Johnston J. C. (1977). The role of familiar units in perception of words and nonwords. *Percept. Psychophys.* 22 249–261 10.3758/BF03199687

[B66] McClelland J. L. Rumelhart D. E. (1981). An interactive activation model of context effects in letter perception: I. An account of basic findings. *Psychol. Rev.* 88 375–407 10.1037/0033-295X.88.5.375 7058229

[B67] Miller G. A. Bruner J. S. Postman L. (1954). Familiarity of letter sequences and tachistoscopic identification. *J. Gen. Psychol.* 50 129–139 10.1080/00221309.1954.9710109

[B68] Muncer S. J. Knight D. C. (2012). The bigram trough hypothesis and the syllable number effect in lexical decision. *Q. J. Exp. Psychol.* 65 2221–2230 10.1080/17470218.2012.697176 22804754

[B69] Muncer S. J. Knight D. Adams J. W. (2014). Bigram frequency, number of syllables and morphemes and their effects on lexical decision and word naming. *J. Psycholinguist. Res.* 43 241–254 10.1007/s10936-013-9252-8 23613200

[B70] Nation K. Angell P. Castles A. (2007). Orthographic learning via self-teaching in children learning to read English: effects of exposure, durability, and context. *J. Exp. Child Psychol.* 96 71–84 10.1016/j.jecp.2006.06.004 16904123

[B71] New B. Grainger J. (2011). On letter frequency effects. *Acta Psychol.* 138 322–328 10.1016/j.actpsy.2011.07.001 21855049

[B72] Owsowitz S. E. (1963). *The Effects of Word Familiarity and Letter Structure Familiarity on the Perception of Words.* Santa Monica, CA: Rand Corporation Publications.

[B73] Pacton S. Fayol M. Perruchet P. (2005). Children’s implicit learning of graphotactic and morphological regularities. *Child Dev.* 76 324–339 10.1111/j.1467-8624.2005.00848.x 15784085

[B74] Pacton S. Perruchet P. Fayol M. Cleeremans A. (2001). Implicit learning out of the lab: the case of orthographic regularities. *J. Exp. Psychol. Gen.* 130 401–426 10.1037/0096-3445.130.3.401 11561917

[B75] Peereman R. Content A. (1995). Neighborhood size effect in naming: lexical activation or sublexical correspondences? *J. Exp. Psychol. Learn. Mem. Cogn.* 21 409–421 10.1037/0278-7393.21.2.409

[B76] Peereman R. Content A. (1999). LEXOP: a lexical database providing orthography-phonology statistics for French monosyllabic words. *Behav. Res. Methods Instrum. Comput.* 31 376–379 10.3758/BF03207735 10495825

[B77] Perea M. Carreiras M. (2008). Do orthotactics and phonology constrain the transposed-letter effect? *Lang. Cogn. Process.* 23 69–92 10.1080/01690960701578146

[B78] Perruchet P. Pacton S. (2006). Implicit learning and statistical learning: one phenomenon, two approaches. *Trends Cogn. Sci.* 10 233–238 10.1016/j.tics.2006.03.006 16616590

[B79] Pitchford N. J. Ledgeway T. Masterson J. (2008). Effect of orthographic processes on letter position encoding. *J. Res. Read.* 31 97–116 10.1111/j.1467-9817.2007.00363.x

[B80] Pitchford N. J. Ledgeway T. Masterson J. (2009). Reduced orthographic learning in dyslexic adult readers: evidence from patterns of letter search. *Q. J. Exp. Psychol.* 62 99–113 10.1080/17470210701823023 18609393

[B81] Rastle K. Davis M. H. New B. (2004). The broth in my brother’s brothel: morpho-orthographic segmentation in visual word recognition. *Psychon. Bull. Rev.* 11 1090–1098 10.3758/BF03196742 15875981

[B82] Rey A. Dufau S. Massol S. Grainger J. (2009). Testing computational models of letter perception with item-level event-related potentials. *Cogn. Neuropsychol.* 26 7–22 10.1080/09541440802176300 18608320

[B83] Rice G. A. Robinson D. O. (1975). The role of bigram frequency in the perception of words and nonwords. *Mem. Cogn.* 3 513–518 10.3758/BF03197523 24203873

[B84] Rumelhart D. E. Siple P. (1974). Process of recognizing tachistoscopically presented words. *Psychol. Rev.* 81 99–118 10.1037/h0036117 4817613

[B85] Saffran J. R. Aslin R. N. Newport E. L. (1996). Statistical learning by 8-month-old infants. *Science* 274 1926–1928 10.1126/science.274.5294.1926 8943209

[B86] Samara A. Caravolas M. (2014). Statistical learning of novel graphotactic constraints in children and adults. *J. Exp. Child Psychol.* 121 137–155 10.1016/j.jecp.2013.11.009 24495840

[B87] Scheerer-Neumann G. (1981). The utilization of intraword structure in poor readers: experimental evidence and a training program. *Psychol. Res.* 43 155–178 10.1007/BF00309827 7302087

[B88] Seidenberg M. S. (1987). “Sublexical structures in visual word recognition: access units or orthographic redundancy?” in *Attention and Performance, XII: The Psychology of Reading*, ed.Coltheart M. (Hillsdale: Lawrence Erlbaum Associates), 245–263.

[B89] Seidenberg M. S. McClelland J. L. (1989). A distributed, developmental model of word recognition and naming. *Psychol. Rev.* 96 523–568 10.1037/0033-295X.96.4.523 2798649

[B90] Singer M. H. (1980). The primacy of visual information inthe analysis of letter strings. *Atten. Percept. Psychophys.* 27 153–162 10.3758/BF03204304 7375334

[B91] Smith F. (1969). The use of featural dependencies across letters in the visual identification of words. *J. Verbal Learn. Verbal Behav.* 8 215–218 10.1016/S0022-5371(69)80064-2

[B92] Spoehr K. T. Smith E. E. (1975). The role of orthographic and phonotactic rules in perceiving letter patterns. *J. Exp. Psychol. Hum. Percept. Perform.* 1 21–34 10.1037/0096-1523.1.1.21 1141834

[B93] Stone G. O. Vanhoy M. Orden G. C. V. (1997). Perception is a two-way street: feedforward and feedback phonology in visual word recognition. *J. Mem. Lang.* 36 337–359 10.1006/jmla.1996.2487

[B94] Taft M. Krebs-Lazendic L. (2013). The role of orthographic syllable structure in assigning letters to their position in visual word recognition. *J. Mem. Lang.* 68 85–97 10.1016/j.jml.2012.10.004

[B95] Vaid J. Frenck-Mestre C. (2002). Do orthographic cues aid language recognition? A laterality study with French–English bilinguals. *Brain Lang.* 82 47–53 10.1016/S0093-934X(02)00008-1 12174814

[B96] van Kesteren R. Dijkstra T. de Smedt K. (2012). Markedness effects in Norwegian–English bilinguals: task-dependent use of language-specific letters and bigrams. *Q. J. Exp. Psychol.* 65 2129–2154 10.1080/17470218.2012.679946 22554207

[B97] Vinckier F. Dehaene S. Jobert A. Dubus J. Sigman M. Cohen L. (2007). Hierarchical coding of letter strings in the ventral stream: dissecting the inner organization of the visual word-form system. *Neuron* 55 143–156 10.1016/j.neuron.2007.05.031 17610823

[B98] Westbury C. Buchanan L. (2002). The probability of the least likely non-length-controlled bigram affects lexical decision reaction times. *Brain Lang.* 81 66–78 10.1006/brln.2001.2507 12081382

